# Quantitative phenotyping of shell suture strength in walnut (*Juglans regia* L.) enhances precision for detection of QTL and genome-wide association mapping

**DOI:** 10.1371/journal.pone.0231144

**Published:** 2020-04-09

**Authors:** Gina M. Sideli, Annarita Marrano, Sara Montanari, Charles A. Leslie, Brian J. Allen, Hao Cheng, Patrick J. Brown, David B. Neale

**Affiliations:** 1 Department of Plant Sciences, University of California, Davis, CA, United States of America; 2 Plant and Food Research, Motueka Research Center, Motueka, New Zealand; 3 Department of Animal Sciences, University of California, Davis, CA, United States of America; Huazhong University of Science and Technology, CHINA

## Abstract

Walnut shell suture strength directly impacts the ability to maintain shell integrity during harvest and processing, susceptibility to insect damage and other contamination, and the proportion of kernel halves recovered during cracking. Suture strength is therefore an important breeding objective. Here, two methods of phenotyping this trait were investigated: 1) traditional, qualitative and rather subjective scoring on an interval scale by human observers, and; 2) quantitative and continuous measurements captured by a texturometer. The aim of this work was to increase the accuracy of suture strength phenotyping and to then apply two mapping approaches, quantitative trait loci (QTL) mapping and genome wide association (GWAS) models, in order to dissect the genetic basis of the walnut suture trait. Using data collected on trees within the UC Davis Walnut Improvement Program (n = 464), the genetic correlation between the texturometer method and qualitatively scored method was high (0.826). Narrow sense heritability calculated using quantitative measurements was 0.82. A major QTL for suture strength was detected on LG05, explaining 34% of the phenotypic variation; additionally, two minor QTLs were identified on LG01 and LG11. All three QTLs were confirmed with GWAS on corresponding chromosomes. The findings reported in this study are relevant for application towards a molecular breeding program in walnut.

## Introduction

English walnuts grown in the United States are sold in-shell, primarily for export at 234,000 tons per year, and shelled for domestic market at 452,000 tons per year totaling 686,000 tons [1]. The term in-shell refers to uncracked nuts containing the kernels within intact shells. Shelled walnuts undergo cracking and processing, during which the shell is cleaned and removed, and kernels are sorted for size, color and quality. The value of intact kernel halves is substantially greater than smaller kernel pieces. Botanically, walnuts are considered drupe-like, consisting of a fleshy mesocarp, or hull, that upon embryo maturity, dehisces, exposing a hard shell enclosing an embryo which is the edible walnut kernel. The pericarp’s innermost wall, known as the endocarp, contains cells that differentiate and lignify, giving rise to the shell structure.

Shell suture strength is a key component of shell integrity, an important economic trait. Walnut shells need to remain intact during harvest and storage (i.e., during tree shaking, transportation, cleaning, and drying) to exclude dirt, insects, moisture, or other contaminates. Weak sutures are often the major entry points and can allow nuts to crack open during harvest or transport, resulting in crop loss. During walnut processing, nuts with weak sutures crack in the wrong direction, reducing or eliminating recovery of kernel halves [2], which has been shown to have increased incidence in microbes in almond [3] and pecan [4]. Suture strength is influenced by tree age and environmental factors. Suture strength and shell thickness were found to be significantly correlated with broken kernels and insect damage; kernel breakage can result in increased microbial damage and decreased antioxidant capacity [5].

The English walnut improvement program (WIP) at the University of California, Davis was initiated in 1949, has released over 20 cultivars, and is currently one of the most important walnut breeding programs worldwide. Shell suture strength, an important breeding objective, which has a strong genetic component, but also can vary with tree age. As a walnut tree matures, shells thicken and sutures often improve [6]. Researchers in Iran, Turkey and China have identified walnut, hazelnut, and macadamia nut suture strength [7–10] as an important trait for their industries. Current suture strength phenotypic evaluation in the UC Davis WIP is conducted manually by hand-cracking. Genotypes are evaluated on a 1–9 scale for ease of splitting the two halves of the shell or tendency to crack along the suture rather than across the cheek or the shell. Although this method is rapid, and has been utilized for decades to provide useful basic information, it suffers from subjectivity and lacks sufficient repeatability and reliability. Automated measurements using a machine has the potential to reduce error variance and introduce more data points on a continuous scale, thus enabling improved detection for marker trait associations.

Together quantitative trait locus (QTL) mapping and genome-wide association studies (GWAS) can be successful in uncovering the genetic basis of complex traits [11]. GWAS is especially useful in tree crops, where generation of bi-parental populations for QTL mapping takes many years and involves high costs [12,13]. Advantages of QTL mapping include the ability to obtain high statistical power in making genotype to phenotype associations without confounding population structure, and evaluate low frequency alleles in a segregating population. To date, many marker trait association studies have been performed in tree crops such as pear [14], apple [15,16] and almond [17]. Specifically, in walnut, Famula et al. [18] applied association mapping to identify the genetic basis of water use efficiency related traits, while Marrano et al. 2019 [19] used both QTL mapping and GWAS to decipher the genetic control of yield, phenology and pellicle color in the UC Davis WIP.

The objectives of this work were: 1) to develop a method for obtaining quantified measurements of suture strength on a continuous scale by using a texturometer machine, developed initially to characterize food texture [20], to streamline data acquisition and computer software for processing and; 2) to use these measurements for QTL mapping and GWAS to identify marker trait associations. Previous studies have utilized a texturometer in walnut solely for measuring suture strength, however the data has not been applied in breeding populations for estimation of marker-trait associations until now. We have demonstrated for the first time that the use of a machine for measuring suture strength can increase precision in the detection of loci under genetic control of this trait.

## Materials and methods

### Mapping populations

A total of 736 walnut trees derived from 40 F1 families of the UC Davis WIP, with an average of 15 trees per family, were evaluated (**Table 1**). The parental crosses were made between 2006 and 2013. Most male parents were used only once, a few males were used two to four times, and one male, ‘Ivanhoe’ was used 13 times; two males were also used reciprocally as females and vice versa. The largest family (n = 180) was a cross between ‘Chandler’, characterized by strong suture lines, and ‘Idaho’, which has weak suture lines, and it was utilized for the QTL mapping approach. All other families, 25 founder trees, and two cultivars ‘Robert Livermore’ and ‘Vina’, representing diverse germplasm from Afghanistan, China, Japan, and France, were included in the GWAS analysis. The range of ages for trees was 4–14 years, with an average of 7.21 years. Founder trees were grafted on RX1, or VX211 rootstock, clonal Paradox, or Paradox seedling. ‘Chandler’ × ‘Idaho’ F1 trees consisted of a mixture of grafted and seedlings on their own rootstock. The parental trees and seedlings utilized in this experiment were grown in 12 blocks at the UC Davis orchards. The founder trees were spaced 30 feet apart, the ‘Chandler’ × ‘Idaho’ trees were spaced 10 feet apart, while the seedling in all remaining blocks were spaced six feet apart. All blocks were watered with micro-sprinklers.

**Table 1:** Experimental design of seal strength data set.

	No. of individuals		No. of families	No. of Blocks	Ages of tree	No. of years
Manual Evaluation		524	34	7	4–10	2015, 2016
Texturometer—QTL mapping		180	1	1	10–14	2016, 2017
Texturometer—GWAS panel		556	39	12	5–9	2015, 2016
Total Texturometer		736	39	12	4–14	2015, 2016, 2017

Includes *Number of individuals*, *families*, *blocks*, *year of harvest*, *and the range of ages of the trees*. Trees that were evaluated with manual evaluation were also evaluated by the texturometer, not all trees that were evaluated with the texturometer were manually evaluated.

### Harvest and post-harvest storage conditions

Walnuts were hand harvested at maturity between August and October. The ‘Chandler’ × ‘Idaho’ population was collected and phenotyped in 2016 and 2017, while the larger population of trees (556) were collected and phenotyped in 2015 and 2016. Walnuts were considered ‘mature’ when the hull easily separated from the shell. Harvested walnuts were placed in labeled mesh polypropylene bags, and air dried at 21°C for two weeks. The walnuts harvested in 2015 were stored in-shell in bins at -5°C and 81.65% relative humidity for a period of five months before evaluation. During the four months of evaluation, walnuts were stored in-shell in bins at 0°C and 87.90% relative humidity. The 2016 and 2017 harvested walnuts were placed directly in storage at 0°C and 87.90% relative humidity for a period of two months before evaluation and retained there for five months during evaluation.

### Suture strength measurements

**Fig 1:**
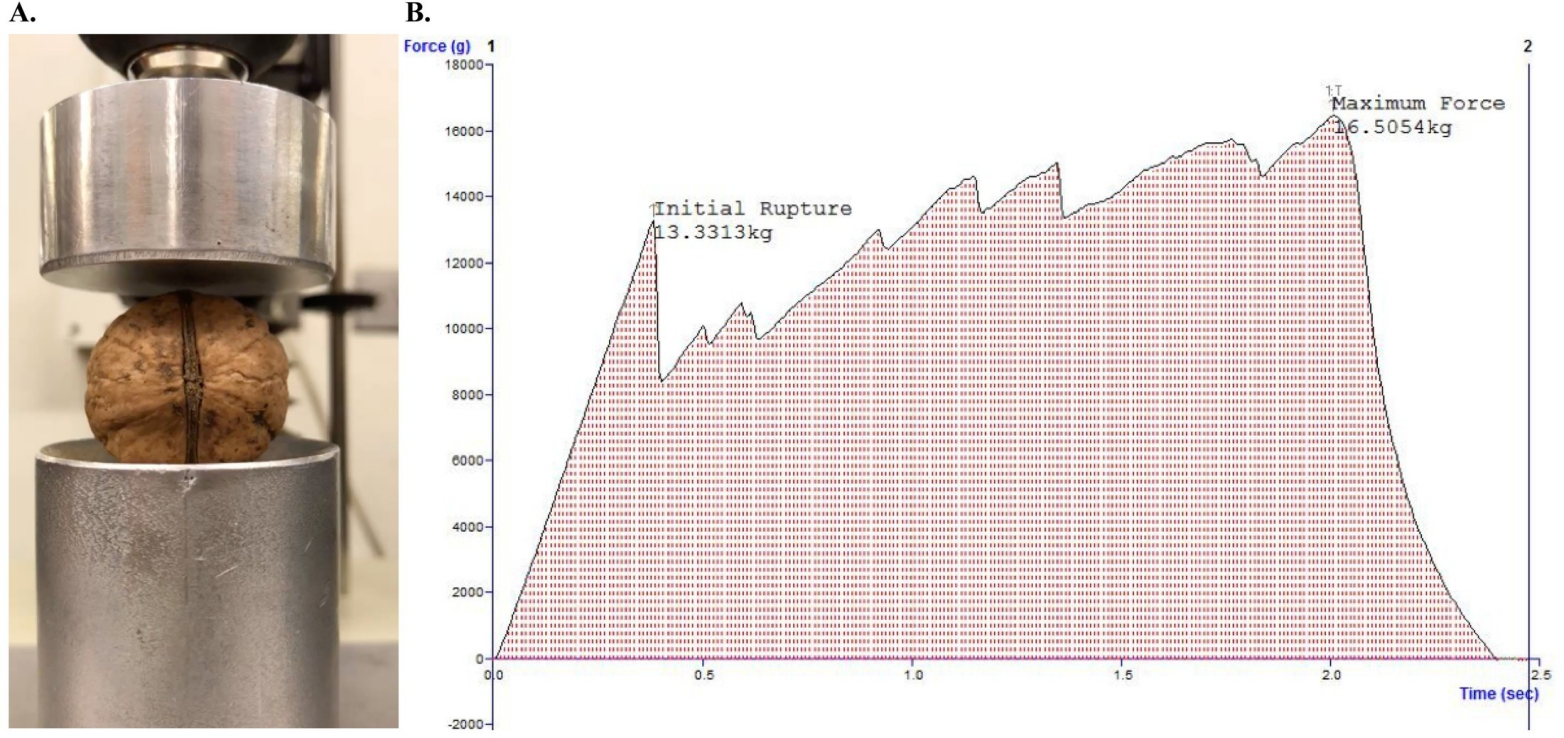
Texturometer phenotyping and graphical output with Exponent software. **A.** position of walnut shell in the texturometer. **B.** *Initial Rupture* is defined as the first crack in the shell, *Maximum Force* is the maximum pressure applied to the shell without destroying the kernel, and the shaded area under the curve is the *Integral*, or total energy exerted during the cracking process.

From each individual tree, 15 walnuts were measured with a texturometer. From the 524 trees not in the ‘Chandler’ × ‘Idaho’ family, ten nuts per tree were cracked by hand with a hammer and scored for ease of separating the shell along the suture line using an interval scale from 1 to 9, where 1 = open suture, 3 = very weak suture, 5 = moderately strong, and 9 = suture much stronger than surrounding shell [21]. With the texturometer (model TA-XT2; Texture Technologies; Surry, England and Exponent 6.1.8.0 software (Stable Micro System; London, UK), the application of force (kg) and time (seconds) to rupture the walnut suture was automatically recorded with the input of a human user. The texturometer has three main components: a stationary cylindrical platform, a moving probe, and a driving unit. Each walnut was positioned on the platform, suture side longitudinally to the moving probe, and subsequently pressed by the probe to a depth of 4.0 mm (**Fig 1**). For an individual tree, walnut shells that were sizably smaller than the others were discarded; in addition, dried, desiccated kernels were discarded from the dataset (**S1 Fig**). The application of force and the time required to deform the walnut shell were continuously recorded over a span of seconds. From these data points, using a custom macro, the integral of the force (total energy), the initial rupture, or first crack in the shell, and the maximum force (i.e. the maximum amount of pressure the shell could withstand without collapsing completely) were measured **(Fig 1)**. Additionally, the texturometer was set as follows: pre-test speed 1.0 mm/sec, test speed 2.00 mm/sec, post-test speed 10.0 mm/sec, trigger force 0.005kg, return to start distance 4.0 mm, calibrated with 2.0 kg weight (**S1 Table**). Basic statistics (means, standard deviations, variances), analysis of variance, and a simple linear regression were performed in R using packages ‘car’, ‘lme4’, ‘agricolae’, ‘ggplot2’, ‘lsmeans’ and ‘lmertest’ to compare the texturometer method with current manual evaluation-method. The Shapiro-Wilks normality test was performed to test normality of phenotypic data.

### Variance component estimates

The ASReml-R 3 [22] software tool was used to estimate variance components from the phenotypic data of 736 trees and a pedigree that was reconstructed with SNP-based data [23]. The linear mixed model utilized for both the texturometer and manual evaluation were as follows:

y=μ+year+a+e

where *y* was the record of the individual tree, μ was the overall mean, *a* was the additive genetic value, and *e* was the error term. Year was fit as the only significant fixed effect, while the additive genetic values were fit as the only random effect. When considering only the texturometer methods, male parent, block, and year were added as fixed effects, and the additive genetic value was set as random. A conditional Wald F-test was used to test the significance of fixed effects in the model, and log-likelihood ratio test was used to choose the best model. A bivariate model was used to estimate the covariance of texturometer trait measurements to both manual evaluation and to Julian harvest date. A repeatability model was applied to assess the repeated observations that were acquired in the two-year dataset with all the same factors (year as fixed) as in the univariate and bivariate model. Narrow-sense heritability was calculated as defined: *h*^2^ = *Va/Vp*, the additive genetic variance divided by the phenotypic variance.

For each individual tree, the mean of the texturometer data points was calculated on the 15 walnut measurements/tree. In order to obtain one value per individual for both years of data collection, the adjusted means were determined using the R package ‘lsmeans’, with block and year as fixed factors in the model. Phenotypic data was analyzed for each year separately and the adjusted means were calculated for both years.

### Genetic map construction

Using the custom Axiom *J*. *regia* 700K SNP Array [23], 339K polymorphic, high quality (Polyhigh resolution) SNPs were used for analysis. Segregating SNPs were then selected which fit into a double pseudo-backcross type, with one parent being heterozygous and the other parent being homozygous (AB x AA/BB and AA/BB x AB), and separated into two datasets.

Genetic maps were constructed in R package ASMap [24] utilizing MST (minimum spanning tree) mapping algorithm [25] to assign markers into linkage groups (LGs) and order them. Distorted SNPs (p-value < 0.01) and markers with missing rate (> 0.10) were removed. Co-mapping SNPs were removed, retaining only one marker per locus. Genetic distance was estimated using the Kosambi mapping function.

Linkage groups were oriented and numbered according to the SNP physical locations onto the new chromosome-level assembly of the *J*. *regia* ‘Chandler’ reference genome v.2.0 (available at https://www.hardwoodgenomics.org/Genome-assembly/2539069).

### QTL mapping

QTL mapping was performed using the R package ‘R/qtl’ [26]. Simple interval mapping was performed with a genome scan utilizing a single QTL model (*scanone*) with both maximum likelihood and “Haley Knott” regression algorithms [27]. Bayes 0.95 credible interval was implemented to locate confidence interval around the most significant SNP. Significance LOD thresholds at ρ = 0.05 were determined by permutation tests with 1,000 permutations. Two-dimensional, two QTL scans were performed with (*scantwo*) in order to assess interactions and possible linkage of multiple QTLs. Interval mapping results were then compared with multiple QTL (MQM) [28] mapping algorithm, which utilizes augmentation for missing data, multiple regression, backward elimination, and selection of significant SNPs outside of interval as a covariate. Composite interval mapping was then performed to compare results among mapping methods; the marker closest to the marker with highest LOD score was added as a covariate, and “Haley Knot” regression algorithm was run in the model [27]. To estimate the phenotypic variance explained by each significant SNP, total variance in the model, and significant SNPs allelic effects, an ANOVA was fit with either a multiple QTL model, if there were two QTLs, or a single QTL model with function *fitqtl*. To adjust QTL locations in a multiple QTL model, the function *refineqtl* was utilized.

### Data preparation for GWAS

Quality control of SNPs was performed prior to GWAS. Using PLINK 1.9 [29], only SNPs with minor allele frequency (MAF) > 0.05, genotypic call error rate > 0.05, and which were in Hardy Weinberg equilibrium (p-value <0.001) were retained. Additionally, individuals with values of heterozygosity above or below the mean of all genotypes ± 3 standard deviations were removed.

In order to evaluate the structure of the mapping population, principal component analysis (PCA) was utilized. Different filtering parameters were applied from those described above: Poly High Resolution SNPs generated with the Axiom *J*. *regia* 700K SNP Array [23] were discarded if they had missing rate > 0.20 and MAF < 0.05; the remaining SNPs were then pruned to be in linkage disequilibrium (LD) > 0.25) using the R package ‘SNPRelate’ [30]. A scree plot was used to assess how many PCs to include as covariates in GWAS analysis.

### GWAS analysis

The estimated breeding values that were generated from variance components calculations in R-ASReml 3 [22] were used for GWAS. Genome-wide associations was conducted by using the Fixed and Random Model Circulating Probability Unification (FarmCPU) algorithm [31] and multi-locus mixed linear model (MLMM) algorithm [32] in R package GAPIT 2 [33]. The MLMM is a multi-linear model (MLM) [34] where both Q (population structure) + K (kinship matrix) are fitted to the model as random effects, reducing type I errors due to spurious associations from relatedness and population structure. A 5% Bonferroni threshold was used to assess significance, and Q-Q plots and Manhattan plots were inspected for evidence of inflation. A multiple corrections test was then utilized to assess SNPs at a less stringent threshold. The number of PCs to add as covariates in the multivariate model was defined using the function model selection implemented in GAPIT, once the initial PCA and scree-plot was evaluated for the maximum number of PCs to add. In particular, FarmCPU implements a generalized linear model (GLM) where PC’s are added as covariates to first scan for single significant markers. Subsequently, markers are in binned into pseudo quantitative trait nucleotides (QTNs), log likelihood estimates are then derived from a random effect model, and best QTNs are set as covariates for another genome scan where the process is repeated until the same QTNs display significance. The GLM model was set to perform 10 iterations, with three PC’s (fourth PC was not informative), a MAF threshold of 0.05, and the default parameters for bin size.

### Data visualization

Mapchart v2.32 was used for visualization of QTL maps [35]. R package MareyMap 1.3 [36] was utilized to visually compare genetic map distance (cM) to physical distance. R package ‘genetics’ was used to format data and ‘LDheatmap’ [37] was used to visualize the LD between significant SNPs.

### Candidate gene analysis

Blocks of linkage disequilibrium around the most significant trait-associated loci were defined with Haploview 4.2 using the default algorithm of [38], where LD blocks are grouped based on 95% of SNP comparisons to be in strong LD (> 0.80). These LD blocks were used to search for candidate genes using the NCBI RefSeq *J*. *regia* database mapped onto the new chromosome-level assembly of the *J*. *regia* ‘Chandler’ reference genome v2.0.

## Results and discussion

### Analysis of variance for texturometer traits

For the two-year dataset (2015–2016) on the 526 individuals, the proportion of within family variance for integral measurements was determined to be 22%, while the proportion of among family variance was 77%. With inclusion of the ‘Chandler’ × ‘Idaho’ dataset, the proportion of within family variance decreased to 19.21%, and the proportion of among family variance increased to 80.64%. Suture strength phenotypic data collected in 2015, 2016 and 2017 were found to be significantly different from each other (ANOVA, “Tukey” ρ < 0.001). The data was not normally distributed, according to the Shapiro-Wilks normality test (ρ < 0.05), and outliers were removed from further analysis. For the 24 families that contained more than 10 individuals, family 11–011 (95-026-16 × 03-001-3382) had the highest Least Square Mean (LSM) score (38.697 ± 0.886; n = 11), while family 11–029 (03-001-665 × 01-007-2) had the lowest LSM score (24.28 ± 0.886; n = 12). The ‘Chandler’ × ‘Idaho’ family walnut shells displayed a great amount of diversity in size and shape (**S1 Fig**). Age and block were not significant factors in the linear model when estimating ANOVA, while year of harvest was a significant factor.

### Variance components, heritability estimates and correlation between traits

With the manual evaluation method, additive genetic variance was found to be lower than environmental variance, which corresponded to its low narrow-sense heritability of 0.16 (**Table 2**). For texturometer-based methods, the additive genetic component for initial rupture, integral and maximum force was much higher than environmental variance, and therefore high heritability was observed for all traits (0.82–0.84; **Table 2**). When considering block as a random effect, the proportion of block variance was estimated to be 9.75%, and when estimating family or male parent effects, the proportion of family variance was found to be 1.96% while the proportion of male parent variance was 13.11%. For the manual evaluation, the proportion of block variance was not significant (ρ > 0.05), and therefore it was not considered in the model. The estimated breeding value coefficient of variation for texturometer was found to be about ten times higher than that of the manual evaluation method (**Table 2**). When considering only ‘Chandler’ × ‘Idaho’ mapping population (n = 180) the environmental variance increased to 33–40% for texturometer phenotypes and therefore narrow-sense heritability estimates were lower than the individuals in the breeding program (**S2 Table**). For the ‘Chandler’ × ‘Idaho’ dataset, year and age of tree were significant factors in the model. When performing the analysis on all individuals including the Chandler’ × ‘Idaho’, narrow-sense heritability for texturometer phenotypes (0.79–0.81) was higher than when only considering the mapping population (**S3 Table**).

**Table 2:** Comparison of manual evaluation vs texturometer measurements.

	N	EBV μ	EBV σ	EBV CV	V_A_	V_R_	h^2^	r
Manual Evaluation	464	4.86	0.11	2.32	0.02 (15.88%)	0.14 (84.12%)	0.16	0.09
Initial Rupture	464	23.58	6.50	27.58	74.49 (82.02%)	16.33 (17.98%)	0.82	0.76
Integral	464	29.60	6.01	20.32	60.41 (81.80%)	13.44 (18.20%)	0.82	0.76
Maximum Force	464	25.28	6.09	24.10	64.41 (83.77%)	12.48 (16.23%)	0.84	0.78

Measurements taken with harvested walnuts in 2015 and 2016. Summary statistics and variance components estimated: *EBV* μ = mean, *EBV σ =* standard deviation, *EBV CV =* coefficient of variation, *V*_*A*_ *=* additive genetic variance, *V*_*R*_ *=* residual genetic variance, *h*^*2*^ *=* narrow-sense heritability, *r =* repeatability.

**Fig 2:**
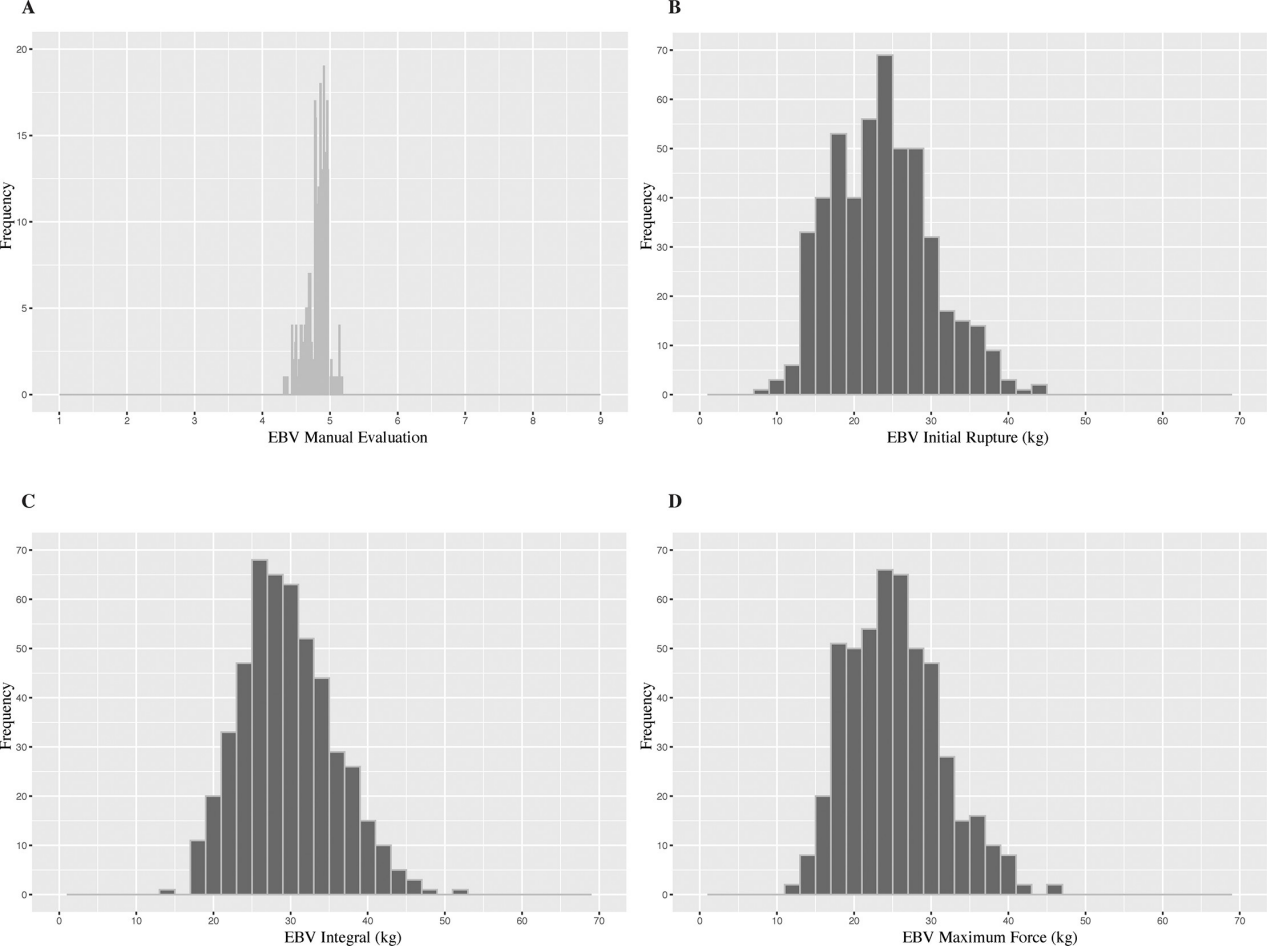
Histogram of estimated breeding values of manual evaluation and texturometer phenotypes. **A.** *Manual Evaluation* based on a scale of 1–9, n = 494, Texturometer phenotypes were measured as kilograms of force, n = 802 (736 with 66 parents). **B.** *Integral*, **C.** *Initial Rupture*, **D.** *Maximum Force*.

Estimated breeding values (EBVs) of top 20 individuals with highest integral values (higher values indicate stronger suture) are displayed in (**S4 Table**); cultivars Chandler, Vina, Hartley, Tulare, and S. Franquette had lower breeding values for the integral trait than for the seedlings in the UC Davis WIP. Individuals from the families 11–011 (95-026-16 × 03-001-3382), 10–016 (00-005-44 × 03-001-2357), 09–028 (95-027-38 × 95-007-13) and 11–030 (04-003-107 × Ivanhoe) were found (**S4 Table**) among the top 20 individuals for suture strength. Estimated breeding values based upon manual evaluation resulted in categorical data that was restricted to scores between 4.3 and 5.3 (**Fig 2**), while for the texturometer phenotypes, the EBVs were continuous and normally distributed on scale between 10–45 kg of force applied (**Fig 2**).

There was high correlation (r^2^_G_ > 0.60) between integral and manual evaluation initial rupture and maximum force, while harvest date and integral were poorly correlated (r^2^_G_ = 0.07, **S5 Table**).

### Genetic map and QTL detection

The ‘Chandler’ genetic map was arranged on 16 linkage groups with a total of 1,165 markers, a total length of 998.31 cM, and an average distance between markers of 0.9 cM (**S1 Fig**). The ‘Idaho’ genetic map consisted of 1,753 markers for a total length of 1,693.88 cM and an average distance between markers of 0.70 cM (**S1 Fig**).

**Fig 3:**
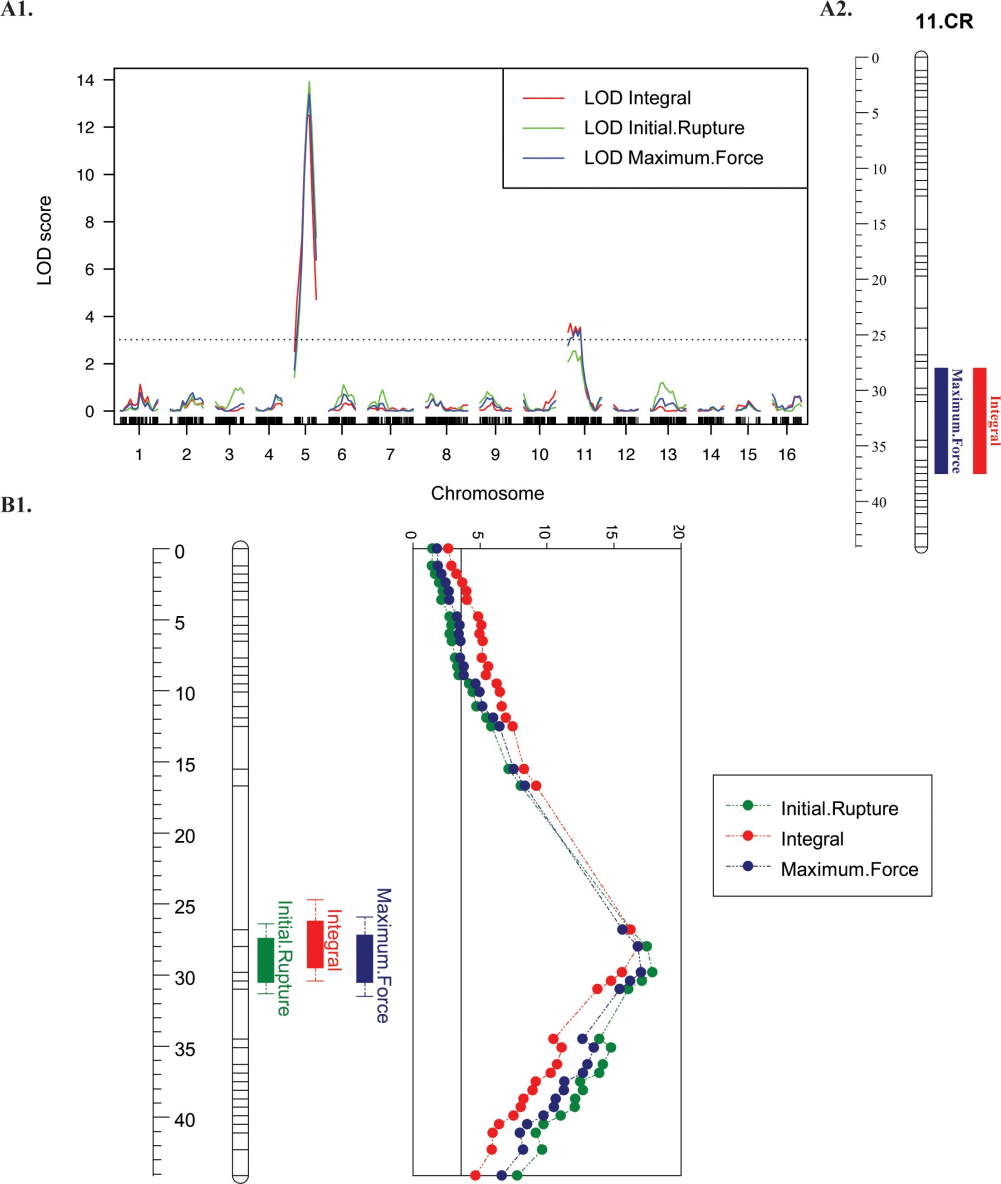
‘Chandler’ QTL mapping for texturometer phenotypes. **A1.** ‘Chandler’ genome wide QTL (MQM) results for Initial Rupture, Integral and Maximum Force phenotypes for the adjusted mean for 2016, 2017. **A2.** LG11 QTL with intervals for Integral and Maximum Force (2016, 2017). **B1.** LG05 major QTL with intervals and LOD graph for Initial Rupture, Integral and Maximum Force (2016, 2017).

**Fig 4:**
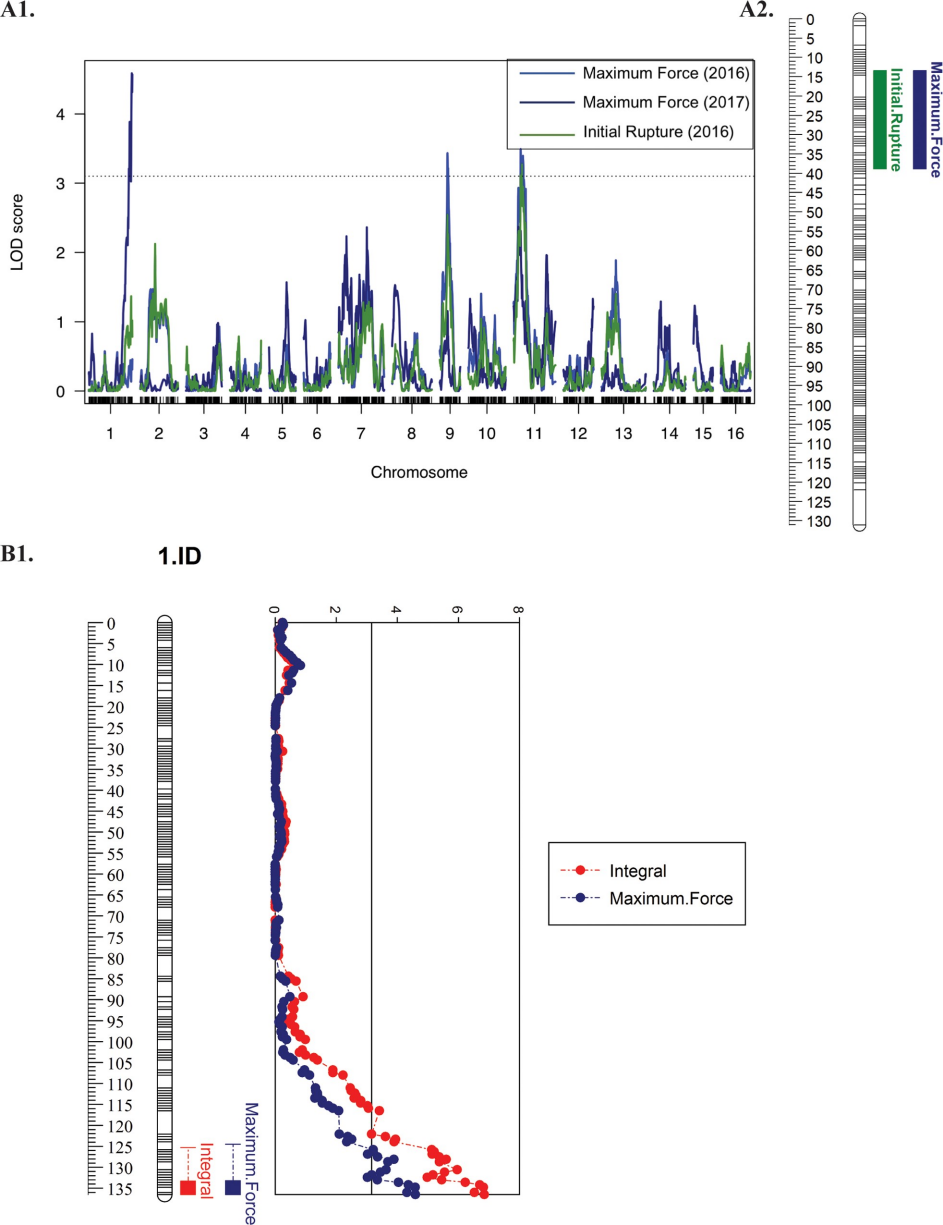
‘Idaho’ QTL mapping of texturometer phenotypes. **A1.** ‘Idaho’ genome wide QTL mapping for Maximum Force (2016, 2017) and Initial Rupture (2016) on LG01, LG09, LG11. **A2.** ‘Idaho’ LG11 with Initial Rupture 2016 and Maximum Force intervals for 2016/2017. **B1.** ‘Idaho’ LG01 for Integral (2016, 2017) and Maximum Force (2017) phenotypes drawn with LOD scores.

Genome-wide thresholds resulted from 1,000 permutation test were 3.08, 3.04 and 3.07 LOD for initial rupture, integral, and maximum force respectively. Using both simple interval mapping (SIM) regression algorithm and MQM mapping, five QTLs in total were detected for the three texturometer-based measures on LG05 and LG11 of ‘Chandler’, explaining 1.9–25.8% of the phenotypic variation (**Fig 3, Table 3**). Five QTLs were also detected on LG1, LG09 and LG11 of ‘Idaho’, explaining 3.86–17.12% of the phenotypic variation (**Fig 4, Table 3**). In addition, the CIM algorithm detected the same QTLs in which the SIM and MQM algorithm detected (S4 Fig, S5 Fig).

**Table 3:** QTL mapping results for texturometer phenotypes in ‘Chandler’ and ‘Idaho’.

**A.**	**Initial Rupture**						
	*QTL*	*Loc (cM)*	*Physical pos (bp)*	*Interval (cM)*	*LOD*	*R*^*2*^ *(%)*	Detected *(Year)*
**‘CR’**							
Chr05	AX.171024351	29.77	12,558,173	27.99–37.52	17.9	25.78	16, 17, 16/17
**‘ID’**							
Chr11	AX.170873965	26.26	7,954,664	13.39–38.88	3.27	8.631	16
**B.**	**Integral**						
	*QTL*	*loc (cM)*	*Physical pos (bp)*	*Interval (cM)*	*LOD*	*R*^*2*^ *(%)*	Detected *(Year)*
**‘CR’**							
Chr05	AX.171164993	27.99	10,015,707	27.99–37.52	17.55	34.20	16, 17, 16/17
Chr11	AX.171167592	24.54	22,728,255	0.0–27.52	5.09	8.29	16, 17, 16/17
Total						44.65	
**‘ID’**							
Chr01	AX.170557708	136.54	44,984,959	124.00–136.55	6.84	17.12	16, 17, 16/17
**C. **	**Maximum Force**						
	*QTL*	*loc* *(cM)*	*Physical pos (bp)*	*Interval (cM)*	*LOD*	*R*^*2*^ *(%)*	Detected *(Year)*
**‘CR’**							
Chr05	AX.170639921	29.77	10,937,209	27.99–37.52	18.07	35.17	16, 17, 16/17
Chr11	AX.171167592	24.54	22,728,255	0.0–25.14	4.59	7.57	17, 16/17
Total						45.13	
**‘ID’**							
Chr01	AX.170557708	136.54	44,984,959	124.00–136.55	4.59	3.86	17
Chr09	AX.170681493	23.82	13,943,092	7.14–37.51	3.42	6.64	16
Chr11	AX.171480798	22.05	4,967,174	13.39–38.88	3.38	8.94	16, 16/17
Total						18.27	

Data collected in 2016, 2017 for 180 individuals in the ‘Chandler’ x ‘Idaho’ population. *‘CR’* = Chandler, *‘ID’* = Idaho, *loc* = location in cM, *LOD* = log of odds score, *Detected Year* = 16/17 is the adjusted mean for both years. If detected in both years, LOD and R^2^ taken from the adjusted mean.

### Principal component analysis

**Fig 5:**
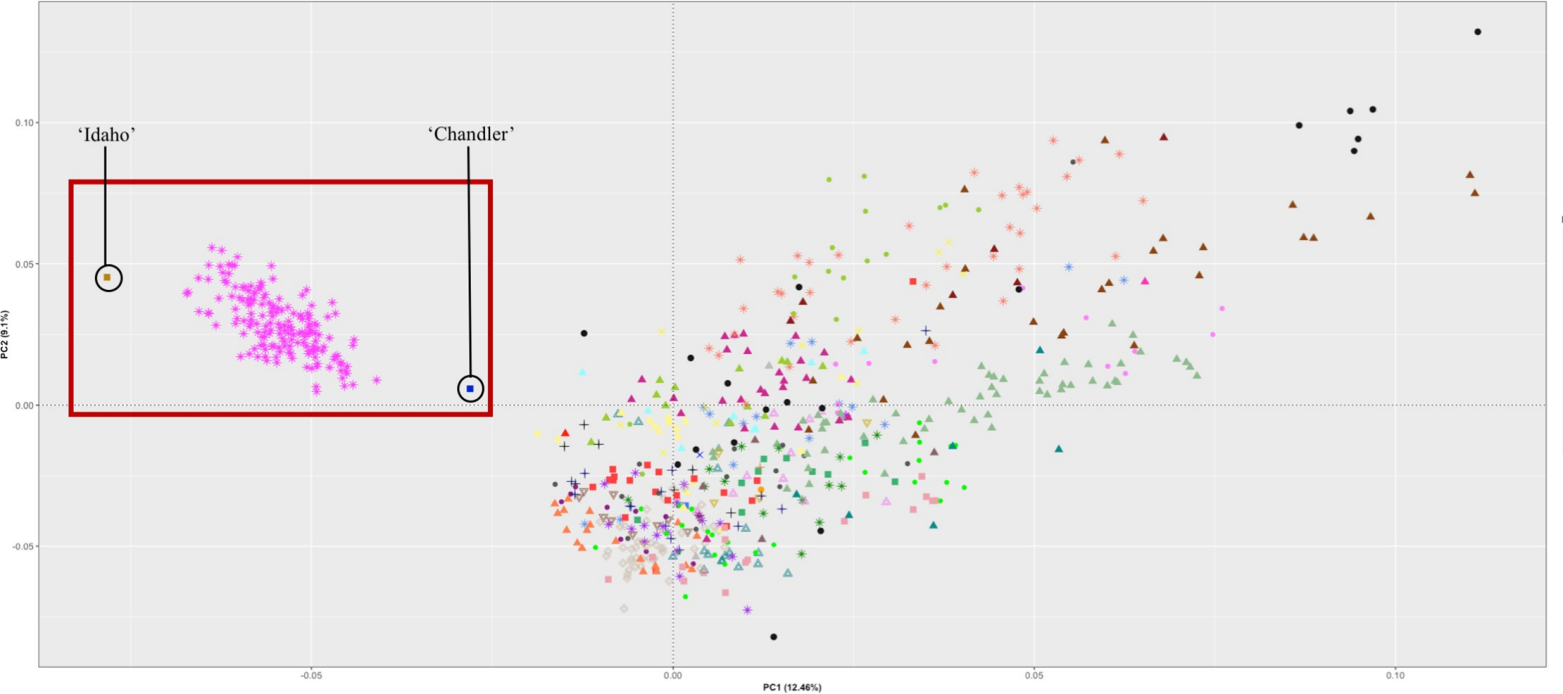
Principal component analysis with 730 individuals. Analysis performed in R-Package SNPrelate with 45,441 SNP markers representing 40 full-sib families. ‘Chandler’ × ‘Idaho’ mapping population highlighted. Each color/shape represent a different family, and the founders of the UC Davis WIP founders are displayed with black circles.

The total amount of SNPs utilized for PCA after filtering was 45,441. By looking at the entire dataset of 730 individuals of the UC Davis WIP, PC1 accounted for 12.46% of the genetic variation and PC2 for 9.10% of the variation. The ‘Chandler’ × ‘Idaho’ population is quite distinct from the other individuals of the breeding program, and the founders are on the opposite side of each other along the PC1. Parents of the other families are dispersed across the PCA plot (**Fig 5**).

### Genome-wide association study

Sixteen significant genotype-to-phenotype associations were detected in total (**Table 4**). Two significant associations were determined on Chr05 for the manual evaluation phenotype, which were in moderate LD and 300 kb away from each other (r^2^ = 0.501, D’ = 1.0); the MLMM detected association was in high LD with the locus associated with the integral phenotype 1.86 Mbp away, (r^2^ = 0.814, D’ = 0.973).

**Table 4:** GWAS results with MLMM and FarmCPU models run with 528 individuals.

Trait	Model^a^	SNP	Chr	Position (bp)	*p* value	Allelic Effect	R^2^^b^	MAF^c^	Allele	Annotation
ME	MLMM	AX. 171135430	5	12647620	3.81^−08^	-0.01	0.09	0.26	A/G uncharacterized LOC109018872*	
ME	FCPU	AX. 170748528	5	13023760	1.18^−16^	-0.08	0.01	0.15	A/G	lamin-like protein*
IR	MLMM	**AX. 171524856**	**5**	**14333096**	1.52^−19^	+0.53	0.08	0.30	A/C	protein FAR1-RELATED SEQUENCE 5-like
IR	FCPU	*AX*. *171577297*	*5*	*14602000*	1.53^−19^	-3.91	0.08	0.29	A/G	uncharacterized mitochondrial protein AtMg00310-like
IR	FCPU	AX. 170923201	1	42331578	1.40^−08^	-1.43	0.01	0.32	T/G	probable WRKY transcription factor 21*
IR	FCPU	AX. 171511499	11	13714234	6.79^−07^	-2.05	0.003	0.36	C/G	leucine-rich repeat receptor-like protein kinase PXL1
I	MLMM	AX. 171535400	5	14512735	6.66^−18^	+0.93	0.03	0.29	A/T	uncharacterized mitochondrial protein AtMg00310-like
I	FCPU	AX. 171007298	5	12933466	5.72^−21^	-3.67	0.03	0.30	A/G	uncharacterized LOC108997528
I	FCPU	AX. 170916442	1	41423083	4.12^−12^	-1.58	0.01	0.42	T/C	N-alpha-acetyltransferase 35, NatC auxiliary subunit
I	FCPU	AX. 170916442	1	41423083	4.12^−12^	-1.58	0.01	0.42	T/C	probable glycosyltransferase AT5g20620
I	FCPU	AX. 170721959	11	13213747	5.12^−10^	+3.21	0.01	0.10	A/G	uncharacterized LOC109000358
MF	MLMM	**AX. 171524856**	**5**	**14333096**	2.12^−20^	+0.53	0.08	0.30	A/C	protein FAR1-RELATED SEQUENCE 5-like
MF	FCPU	*AX*. *171577297*	*5*	*14602000*	5.90^−21^	-3.71	0.08	0.29	A/G	uncharacterized mitochondrial protein AtMg00310-like
MF	FCPU	AX. 170857218	1	37594096	6.38^−06^	+1.41	0.01	0.15	T/G	uncharacterized LOC109010139
MF	FCPU	AX. 171101817	9	12818992	8.07^−09^	-1.27	0.01	0.36	A/G	myb related protein Myb4-like*
MF	FCPU	AX. 171547773	11	10995387	1.25^−09^	-1.59	0.01	0.37	C/G	peptide-N(4)-asparagine amidase

ME = Manual Evaluation, IR = Initial Rupture, I = Integral, MF = Maximum Force. Bold and italicized SNPs indicate same loci across phenotypes. Underlined allele is the effect towards reference or alternate allele. Asterisk* denotes closest gene to physical SNP position.

^a^ Model MLMM multi locus mixed model employed in GAPIT, FARMCPU fixed and random model circulating probability unification.

^b^ R^2^ variance explained for each significant SNP.

^c^ MAF minor allele frequency, threshold set at 0.05.

**Fig 6:**
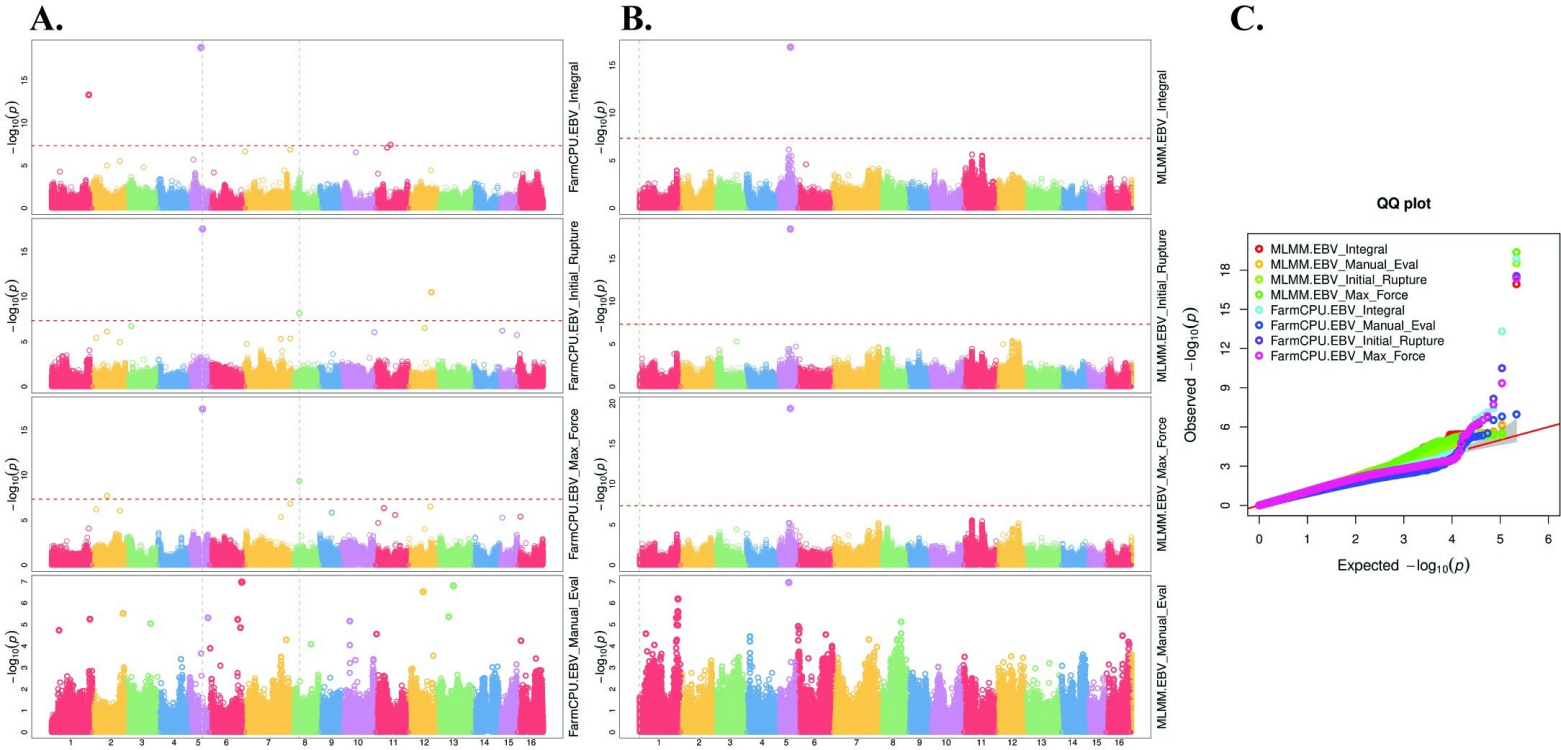
GWAS results displaying Manhattan and Q-Q plots for walnut 16 chromosomes. **A.** FarmCPU model **B.** MLMM model **C.** Multiple Q-Q plots for GWAS analysis of manual evaluation and texturometer traits with MLMM and FarmCPU models.

Utilizing the texturometer phenotypes, five significantly associated SNPs were identified on Chr05 using the MLMM algorithm, and were consistent for all phenotypes and no other associations were determined (**Fig 6**). The significant associations detected with FarmCPU algorithm were consistent with associations detected with MLMM, with the addition of significant loci at or near the threshold for the texturometer methods on Chrs 01, 02, 07, 08, 09, 11, 13.

**Fig 7:**
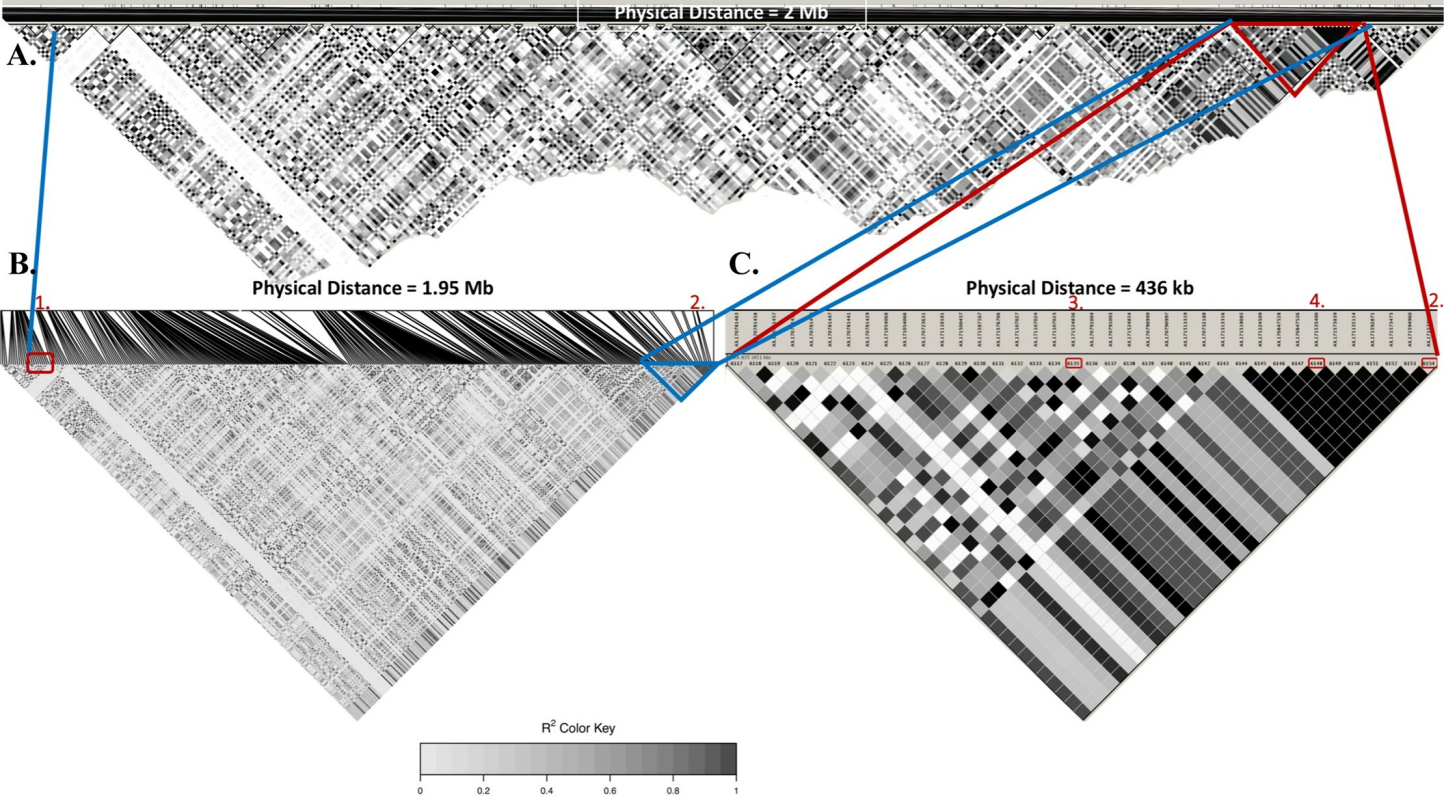
Pairwise linkage disequilibrium plots along Chr05. **A.** Total physical distance shown is 2 Mbp. **B.** Highlighted LD block includes **1.** Manual evaluation QTL (AX.171135430) at 12,647,620 bp and **2.** Maximum Force, Initial Rupture QTL (AX.171577297) at 14,602,000 bp. **C.** LD block (436 kb) with **3.** Maximum Force and Initial Rupture QTL (AX.171524856) at 14,333,096, bp **4.** Integral (AX.171535400) at 14,512,735 bp **2.** Maximum Force and Initial Rupture. Scale 0 is strong evidence for recombination, and dark grey-black represents evidence for strong LD (R^2^).

**Fig 8:**
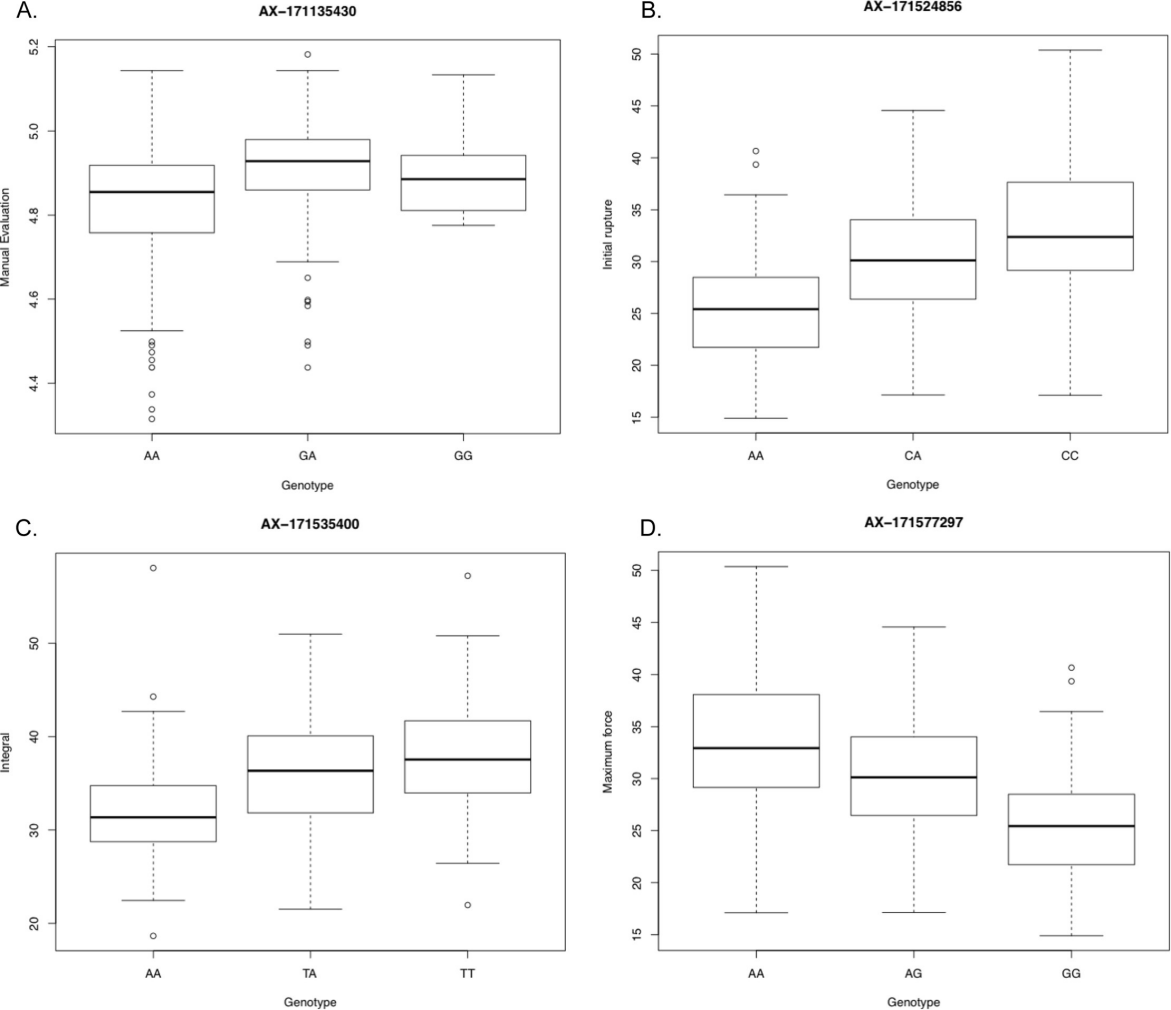
Genotypic class plots for most significant SNPs of each measure. **A.** Manual Evaluation at 13 Mbp, **B.** Initial Rupture at 14.3 Mbp, **C.** Integral at 14.5 Mbp, **D.** Maximum Force at 14.3Mbp.

For Chr05, measures of LD were found to be high between the texturometer methods as they were in the same LD block of 436 kb (**Fig 7**). Both maximum force and initial rupture significant loci were in LD with the two SNPs associated with integral phenotype (r^2^ = 0.991, D’ = 0.995), 179 Kb and 269 Kb away (same LD block of 436 Kb) and even at distances 1.39 Mbp away (r^2^ = 0.982, D’ = 1.0) (**Table 4, Fig 6**). The SNP on Chr05 associated to the manual evaluation trait was found to be in a distant LD block 1.45 Mbp apart from the texturometer LD block (**Fig 7**). Most significant SNPs for both manual evaluation and texturometer phenotypes had negative effects, for the alternative allele (**Fig 8**). Physical positions of the most significant SNPs in GWAS are in overlapping chromosomal regions for QTLs on LG05 and LG11 in ‘Chandler’ (**S3 Fig)**. The same was observed for ‘Idaho’ QTLs found on LG01 and LG11 **(S3 Fig)**. Other co-located SNPs in GWAS and QTL mapping for ‘Idaho’ were on Chr09 and LG09 for maximum force in an overlapping region of the QTL interval.

### Candidate gene analysis

Candidate genes were identified for texturometer phenotypes on Chr05. FAR1 was identified for maximum force and integral. It plays a role in growth and development with light perception capture via phytochrome signaling [39]. Peptide-N(4)-(N-acetyl-beta-glucosaminyl) asparagine amidase gene was also found for the maximum force SNP on Chr11 and aids in the addition of glycans (saccharides) to proteins [40]. Also, on Chr01 the glycosyltransferase gene, which codifies an enzyme critical in establishing structure with glycosidic linkages with sugar transfer [41], was identified for the integral measurement. More specifically, a probable xyloglucan endotransglucosylase gene was found within the initial rupture QTL on Chr05 The function of this protein is to cleave and relegate xyloglucan polymers which are primary constituents in primary cell wall and cell wall growing tissues [42](**S6 Table**).

Suture strength in nuts is poorly understood because there has not been a universal method of measurement. We describe a quantitative phenotyping method for measuring the strength of walnut shell suture and demonstrate its usefulness in improving the ability to discriminate between strong and weak sutured individuals by decreasing the variability inherent in subjective phenotyping.

In line with our texturometer findings, other studies have measured the suture strength of walnut [7,8] and macadamia nut [9] with a universal testing machine, and also defined the maximum force at the position perpendicular to the plane of the suture line, as requiring more energy to rupture the suture, while the minimum force applied was found along the suture line. However, the Turkish walnut cultivars tested by [7] had lower suture strengths than the cultivars and seedlings from the UC Davis WIP(149N along suture, or 15.19 kg/f, and 224N or 22.84 kg/f respectively). The integral measurement accounts for the force and time required for deformation of the shell, and is the total energy exerted or the area under the curve; a shell with a high integral would be one that can withstand the greatest amount of pressure per unit of time.

Narrow-sense heritability is a measure of how strongly the phenotype is correlated with genotype [43]. In our study, the manual evaluation method yielded a low h^2^, while the texturometer method gave a moderately high heritability, and thus more accurately predicts suture strength. In comparing variance components, the texturometer method gave a lower residual error than manual evaluation, indicating reduced phenotyping error. Progress in suture strength over time in the UCD WIP is indicated by breeding values of seedling trees higher than that of the founders.

Our phenotyping method based on a texturometer indicated that the integral measurement can most accurately account for total energy required to rupture the walnut shell suture because it considers the total stress and strain placed upon the suture, rather than force alone. A computerized penetrometer was used similarly in an apple breeding program to accurately select top performing individuals [44], and a digital testing machine was used to phenotypically analyze fruit firmness QTLs for tomato [45]. We successfully demonstrated that the use of a texturometer, for measuring walnut shell suture strength, is more sensitive to capturing variation and therefore can increase the detection of additive genetic component for this trait.

We observed some degree of the Beavis effect [46] between explained phenotypic variance detected for QTL mapping compared to GWAS. Depending upon the power of an experiment, the estimated effects of declared QTL can have an upward bias; the magnitude of effects are inflated for progeny size of 100, somewhat inflated for progeny size of 200, and close to actual effect with progeny size of 1000 [46]. Xu et al. 2003 [47] found that if the sample size was 200 and the estimated effects are greater than 0.10 then the overestimated bias is about 7%. In our QTL mapping study with a population size of about 200, only the QTLs detected on Chr05 had an estimated effect over 10%, while other QTLs explained between 3–8% variation, and in GWAS the average genetic effect was 1%. Therefore, the QTLs detected from QTL mapping are likely to an overestimated bias. For testing and validation of markers for marker-assisted selection, the QTLs with the largest effect should be selected.

Marker-trait associations enabled us to discover, for the first time, the relatively simple control for the suture strength trait in walnut. We performed a combined approach of QTL mapping and GWAS analysis in order to identify specific loci contributing to trait variation. While we observed that the manual evaluation method was able to detect significant loci in GWAS analysis, the significance was just at the threshold line and the SNP was further away from the candidate gene detected with the texturometer methods. The texturometer methods yielded a higher heritability and genetic positions that were in high LD with each other, contributed to greater amount of variance, and were much more highly significant than the manual evaluation.

## Conclusions

These statistical associations are important for the development of molecular markers to be tested and applied in breeding programs, use of which will aid in quick and accurate parental and seedling selection of individuals with appropriate and moderate shell suture strength. The use of machines for phenotyping to replace human measurements can increase the accuracy of breeding in phenotypically based breeding programs. Here we have also shown that quantitative phenotyping was necessary to detect precise marker trait associations. We utilized the power of both QTL mapping and GWAS to determine causative loci for the suture strength trait, which can now enable genomic predictions, and can contribute to marker-assisted development.

## Supporting information

S1 Fig The diversity in walnut shells of ‘Chander’ × ‘Idaho’ cross. **A**. *= 3 suture-lined*, **B**. *= protrusion*, **C**. *= small*, **D**. *= dessicated*, **E**. *= “normal”*.
(TIF)

S2 Fig Linkage groups arranged on chromosomes. **A.** ‘Chandler’ genetic map with 1165 markers. **B.** ‘Idaho’ genetic map with 1753 markers.
(TIF)

S3 Fig Genetic distance plotted against physical position. Positions highlighted red are significant marker-trait associations. **A.** ‘Chandler’ Chr05. **B.** ‘Chandler’ Chr11. **C.** ‘Idaho’ Chr01. **D.** ‘Idaho’ Chr11.
(TIF)

S4 Fig Simple interval mapping compared with composite interval mapping in ‘Chandler’ population. Simple interval mapping displayed in blue, composite interval mapping displayed in red, covariate displayed in green. **A1.** Initial rupture genome-wide scan, **A2.** Initial rupture chromosomes 5 and 11, **B1.** Integral genome-wide scan, **B2**. Integral chromosomes 5 and 11, **C1**. Maximum force genome-wide scan, **C2.** Maximum force chromosome 5 and 11.
(TIF)

S5 Fig Simple interval mapping compared with composite interval mapping in ‘Idaho’ population. Simple interval mapping displayed in blue, composite interval mapping displayed in red, covariate displayed in green. **A1.** Initial rupture genome-wide scan, **A2**. Initial rupture chromosomes 1, 9, and 11, **B1.** Integral genome-wide scan, **B2.** Integral chromosomes 1, 9, and 11, **C1.** Maximum force genome-wide scan, **C2.** Maximum force chromosome 1, 9, and 11.
(TIF)

S1 Table TA-XT2 texturometer settings and macro commands developed in Exponent (Texture Technologies Corp.) software. *Pre-test speed* is the speed of probe prior to test start, *Test-speed* is the speed at which the probe moves for the duration of the test, *Post-test* is the speed at which the probe returns to the start position, **Trigger force** is the resistance at which the probe is sensitive to as it presses the walnut, *Return to start distance* is the amount of compression of the probe to the walnut shell, *Calibration* was performed prior to each set of tests with a 2 kg weight.
(XLSX)

S2 Table Estimated Breeding Values summary statistics and variance components estimates of ‘Chandler’ × ‘Idaho’ mapping population. Harvested data collected in 2016, 2017 for 181 individuals. *EBV* μ mean, *EBV σ standard deviation*, *EBV CV coefficient of variation*, *V*_*A*_ *additive genetic variance*, *V*_*R*_ *residual genetic variance*, *h*^*2*^ *narrow-sense heritability*, *r repeatability*.
(XLSX)

S3 Table Estimated Breeding Values summary statistics and variance components estimates of GWAS panel of individuals and ‘Chandler’ × ‘Idaho’ mapping population. Harvested data collected in 2015, 2016, 2017 for 736 individuals. *EBV* μ mean, *EBV σ standard deviation*, *EBV CV coefficient of variation*, *V*_*A*_ *additive genetic variance*, *V*_*R*_ *residual genetic variance*, *h*^*2*^ *narrow-sense heritability*, *r repeatability*.
(XLSX)

S4 Table Estimated breeding values for the individuals with highest integral values within the WIP. The first 5 entrees are standard cultivars displayed for comparison.
(XLSX)

S5 Table Estimates of genetic correlation between traits using a bivariate model. Covariance components, COV_A_ for each trait were calculated and rescaled to give genetic correlations between traits r^2^_G ._
(XLSX)

S6 Table Gene Annotation products for each significant SNP with QTL mapping and GWAS. *FCPU* is FarmCPU model. Trait abbreviations *I* = integral, *ME* = manual evaluation, *IR* = initial rupture, *MF* = maximum force, *ID* = ‘Idaho’, *CR* = ‘Chandler’, *16* = 2016, *17* = 2017.
(XLSX)

S1 File CRxID_QTL_phenotype. ‘Chandler’ × ‘Idaho’ phenotypic dataset utilized for QTL mapping.
(XLSX)

S2 File Suture_GWAS_phenotype. GWAS panel of individuals within the UC Davis WIP.
(XLSX)
