# Supplementary figures and images for: Quantitative phenotyping of shell suture strength in walnut (Juglans regia L.) enhances precision for detection of QTL and genome-wide association mapping
Source: PLoS One. 2020 Apr 9;15(4):e0231144. doi: 10.1371/journal.pone.0231144 (PMC7144996; doi:10.1371/journal.pone.0231144)

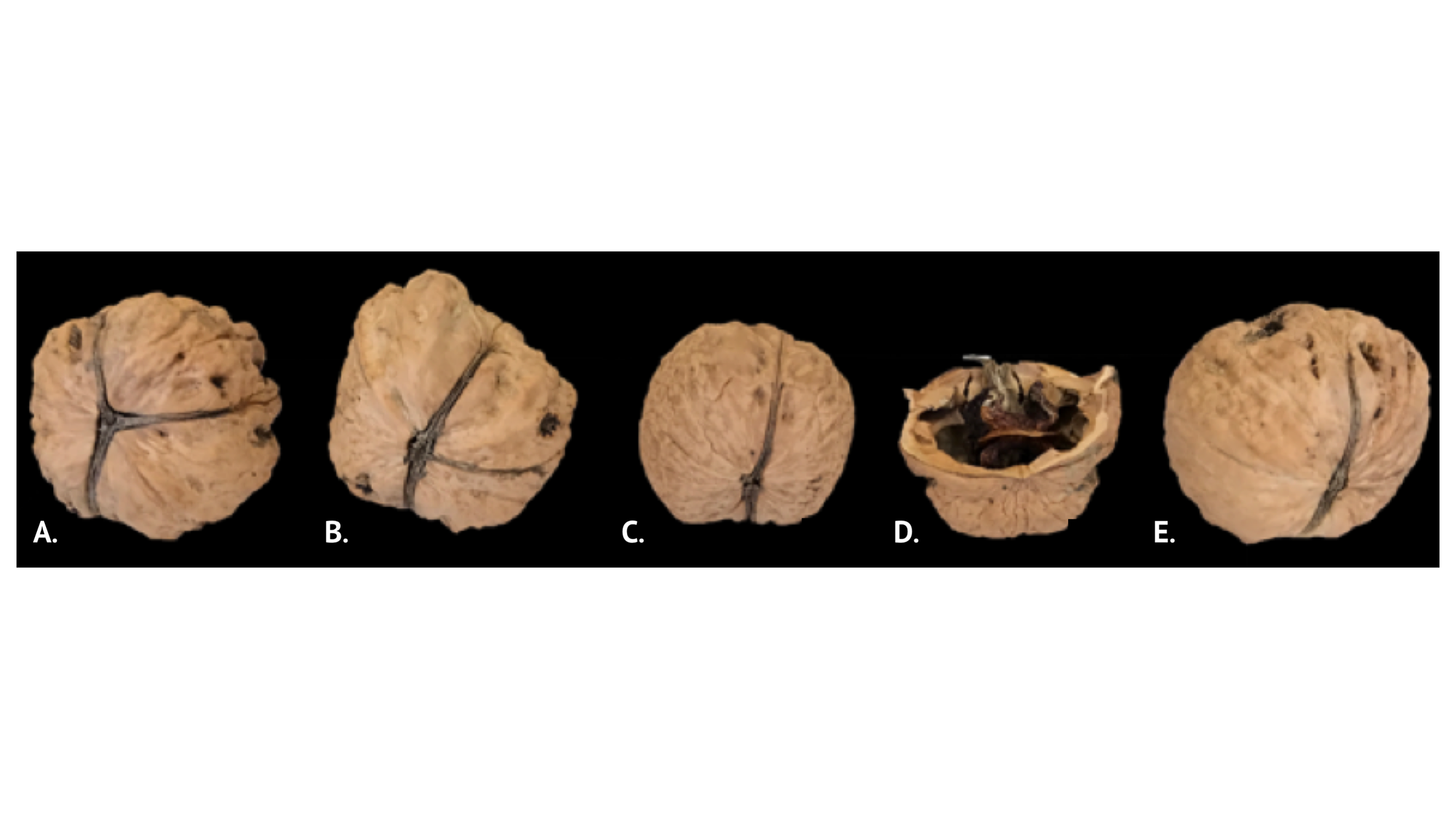

Supplement: S1 Fig — A. = 3 suture-lined, B. = protrusion, C. = small, D. = dessicated, E. = “normal”. (TIF) [file pone.0231144.s001.tif]

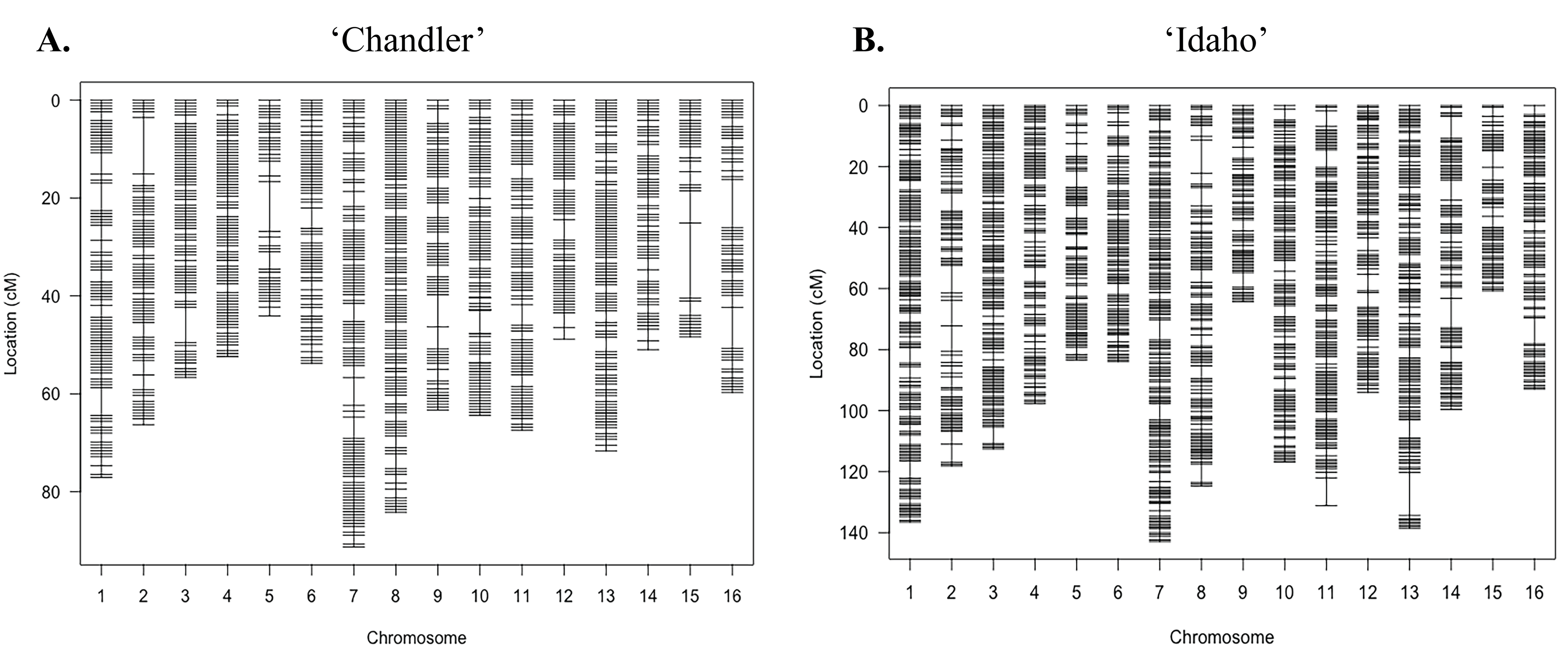

Supplement: S2 Fig — A. ‘Chandler’ genetic map with 1165 markers. B. ‘Idaho’ genetic map with 1753 markers. (TIF) [file pone.0231144.s002.tif]

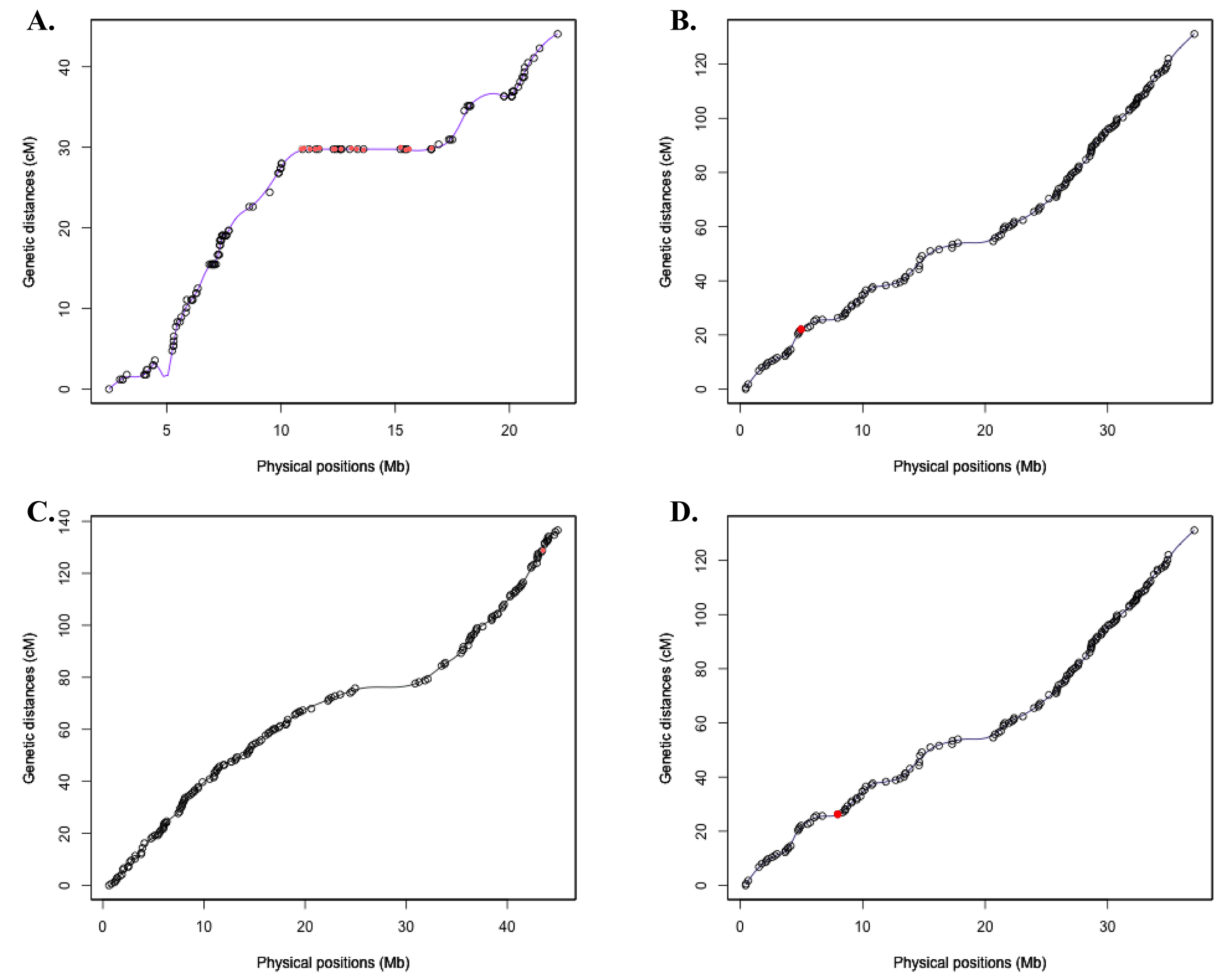

Supplement: S3 Fig — Positions highlighted red are significant marker-trait associations. A. ‘Chandler’ Chr05. B. ‘Chandler’ Chr11. C. ‘Idaho’ Chr01. D. ‘Idaho’ Chr11. (TIF) [file pone.0231144.s003.tif]

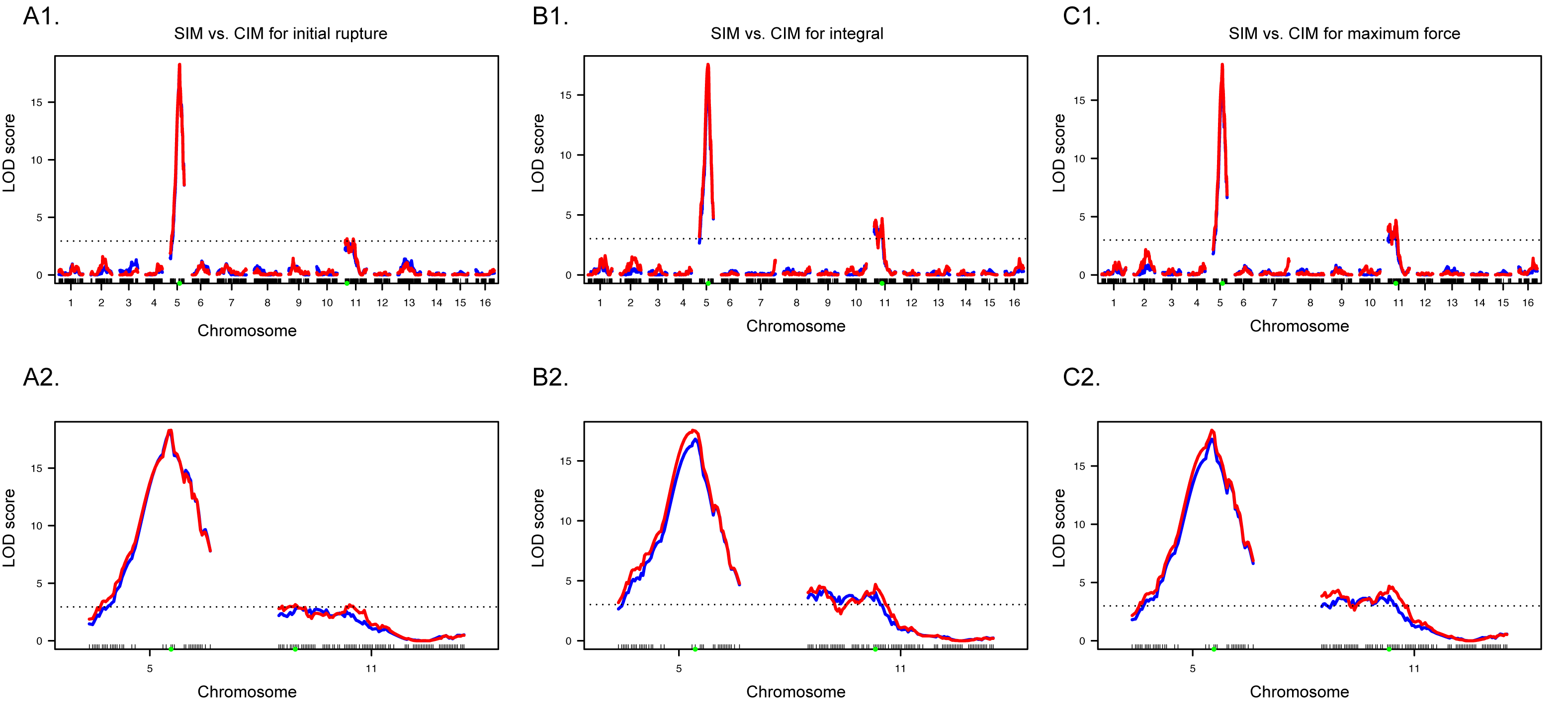

Supplement: S4 Fig — Simple interval mapping displayed in blue, composite interval mapping displayed in red, covariate displayed in green. A1. Initial rupture genome-wide scan, A2. Initial rupture chromosomes 5 and 11, B1. Integral genome-wide scan, B2. Integral chromosomes 5 and 11, C1. Maximum force genome-wide scan, C2. Maximum force chromosome 5 and 11. (TIF) [file pone.0231144.s004.tif]

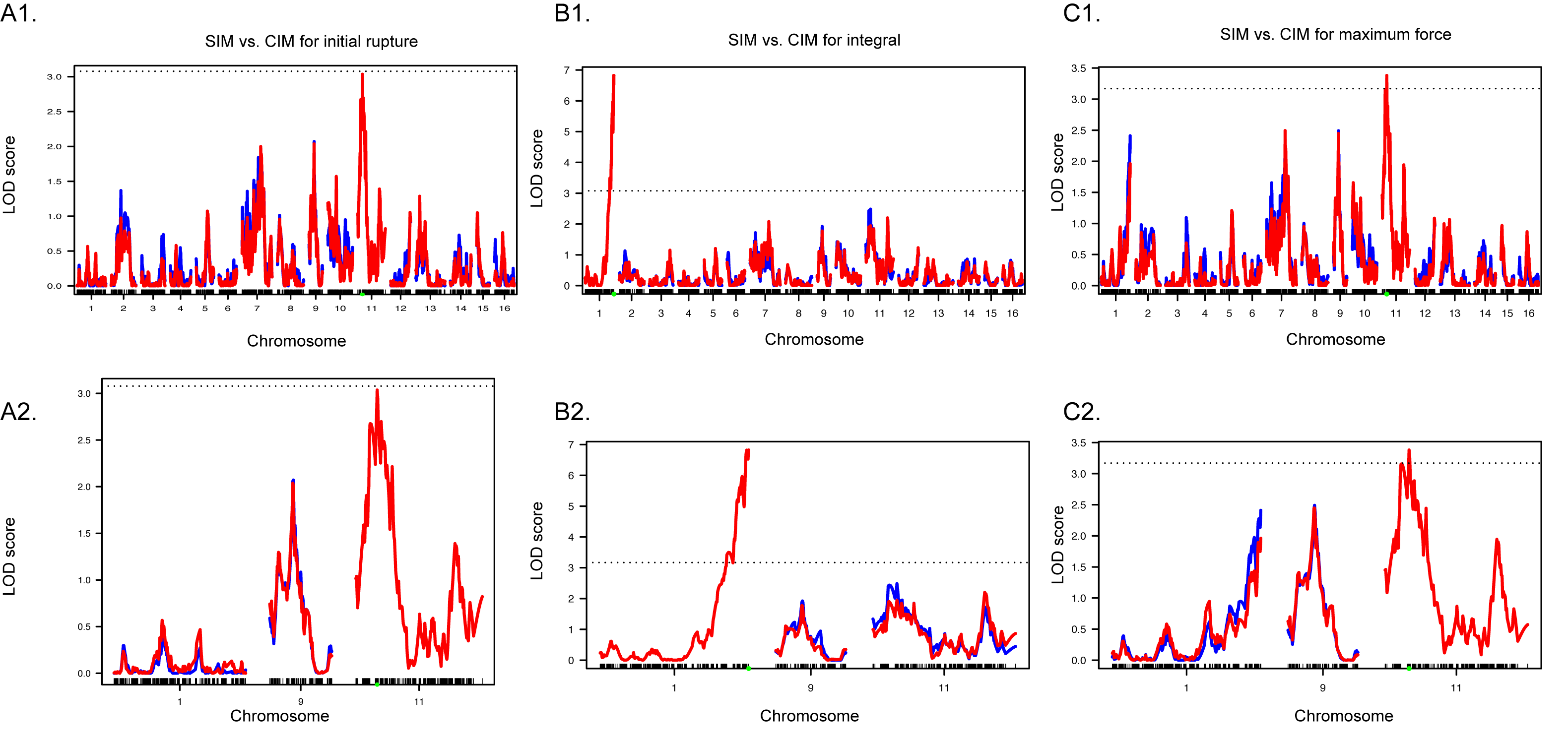

Supplement: S5 Fig — Simple interval mapping displayed in blue, composite interval mapping displayed in red, covariate displayed in green. A1. Initial rupture genome-wide scan, A2. Initial rupture chromosomes 1, 9, and 11, B1. Integral genome-wide scan, B2. Integral chromosomes 1, 9, and 11, C1. Maximum force genome-wide scan, C2. Maximum force chromosome 1, 9, and 11. (TIF) [file pone.0231144.s005.tif]
